# A moderate-carbohydrate diet with plant protein is inversely associated with cardiovascular risk factors: the Korea National Health and Nutrition Examination Survey 2013–2017

**DOI:** 10.1186/s12937-020-00603-2

**Published:** 2020-08-14

**Authors:** Kyungho Ha, Kisun Nam, YoonJu Song

**Affiliations:** 1 Graduate School of Public Health, Seoul National University, Seoul, 08826 Korea; 2 Health & Nutrition Research Center, Pulmuone Co., Ltd., Seoul, 06367 Korea; 3 Major of Food and Nutrition, The Catholic University of Korea, 43 Jibong-ro, Wonmi-gu, Bucheon-si, Gyeonggi-do 14662 Republic of Korea

**Keywords:** Moderate carbohydrate diet, Plant protein, Dyslipidemia, Metabolic syndrome, Korea

## Abstract

**Background:**

Because a moderate-carbohydrate diet reportedly has minimal risks, the substitution of carbohydrate for protein has been emphasized. Few studies have explored the effect of moderate-carbohydrate diets with higher protein intake in Asians, who typically consume a high-carbohydrate low-fat diet. Therefore, this study evaluated the associations of moderate- versus high- carbohydrate diets with cardiovascular risk factors among Korean adults by protein source.

**Methods:**

This study included 7965 adults (3196 men, 4769 women) aged ≥ 19 years who participated in the 2013–2017 Korea National Health and Nutrition Examination Survey. Dietary intake was assessed by a 24-h recall method and four types of diet were defined: a moderate-carbohydrate diet with plant protein (MCP) or animal protein (MCA) and a high-carbohydrate diet with plant protein (HCP) or animal protein (HCA).

**Results:**

Compared with the MCP group, men in the other three groups had significantly higher odds ratios (ORs) for elevated total cholesterol, reduced high-density lipoprotein (HDL)-cholesterol, and metabolic syndrome. Among women, only the HCP group had an increased OR for reduced HDL-cholesterol, compared with the MCP group. Similar associations were observed in younger adults (19–49 years). In addition, younger adults in the MCA group exhibited higher ORs for elevated triglycerides in men and elevated total cholesterol in women, compared with those in the MCP group.

**Conclusions:**

A moderate-carbohydrate diet with a high intake of plant protein was inversely associated with cardiovascular risk factors, especially among younger Korean adults. Further intervention studies are required to confirm this relationship and develop the optimal diet for cardiovascular health in the Korean population.

## Background

In recent decades, research interest in a low-carbohydrate diet versus a low-fat diet has increased due to its health effects. A low-fat diet has been long emphasized to prevent and manage cardiovascular diseases, while a low-carbohydrate diet has an effect on weight loss comparable to a low-fat diet [1, 2]. In terms of cardiovascular risk factors, a low-carbohydrate diet has been associated with a decreased triglyceride level and increased high-density lipoprotein (HDL)-cholesterol level in meta-analyses of randomized controlled trials [3, 4].

There is no universal definition of a low-carbohydrate diet and several types have been formulated [2, 5]. In addition, carbohydrate intake differs among countries and a low-carbohydrate intake can be defined differently by population. For example, the average intake of carbohydrate is approximately 45–50% of energy among adults from the United States (US), United Kingdom, and Canada [6–8], compared with ~ 60% among adults from Korea and Japan [9, 10]. Nevertheless, a low-carbohydrate diet is typically regarded as a carbohydrate intake of less than 45% [11] or 40% [5] of the total energy.

A recent prospective study reported a minimal risk of mortality for a moderate carbohydrate intake (50–55% of energy), while high- and low-carbohydrate diets were related to increased mortality [12]. Additionally, substitution of carbohydrate for animal protein and fat in a low-carbohydrate diet was associated with an increased risk of mortality, compared with substitution of carbohydrate for plant protein and fat [12].

High-protein diets have been a focus with regard to replacing carbohydrate or fat in the diet [13, 14]. Noakes et al. [13] reported that a high-protein diet was associated with a greater reduction in fat mass and body weight compared with conventional high-carbohydrate low-fat diets, undertaken for 12 weeks, among subjects with an elevated serum triacylglycerol level at baseline. Morenga et al. [14] reported that a relatively high-protein high-fiber diet for 10 weeks improved body composition and metabolic risk factors, compared with a standard low-fat high-carbohydrate diet in overweight women.

The source of dietary protein (plant or animal) is important for cardiometabolic health [15, 16]. According to a recent systematic review, soy protein may reduce the risk of cardiovascular diseases such as hypercholesterolemia and hypertension [16]. Similarly, recent longitudinal studies showed that high intake of protein from nuts and seeds in US adults was associated with decreased mortality from cardiovascular disease [17]; moreover, animal protein intake in Australian adults was associated with an increased incidence of metabolic syndrome, whereas plant protein intake showed the opposite association [18]. However, few studies have explored the effect of moderate-carbohydrate diets according to protein source in Asian populations, who typically consume a high-carbohydrate low-fat diet. Thus, this study evaluated associations of cardiovascular risk factors with moderate- and high-carbohydrate diets according to protein source among Korean adults.

## Methods

### Study design and participants

This study used cross-sectional data from the 2013–2017 Korea National Health and Nutrition Examination Survey (KNHANES). KNHANES is a nationwide survey conducted by the Korea Centers for Disease Control & Prevention (KCDC) to evaluate the health and nutritional status of Koreans; it comprises a health examination, health interview, and nutrition survey. To select a nationally representative sample of the Korean population, approximately 10,000 non-institutionalized individuals aged ≥ 1 years are recruited each year, in accordance with a complex multi-stage clustered probability design. The details of KNHANES are available elsewhere [19].

Among 27,220 adults aged 19 years or older who participated in a 24-h dietary recall survey, those who did not have information regarding anthropometric and biochemical measurements such as waist circumference, blood pressure, blood glucose, and blood lipid levels (*n* = 3992); those who had been diagnosed with or were taking medication for diabetes, hypertension, or dyslipidemia (*n* = 8128); those who had been diagnosed with stroke, myocardial infarction, or angina (*n* = 232), those women who were pregnant or lactating (*n* = 225); and those who reported an implausible energy intake (< 500 or > 5000 kcal/day) (*n* = 287) were excluded. A total of 14,356 participants were eligible for this study.

### Assessment of dietary intake

Energy and macronutrient intakes, including intake of dietary fiber, were assessed using a 1-day 24-h dietary recall method. A trained staff member visited the household of the participant and measured all foods and beverages consumed by the participant within 24 h before the day preceding the survey. Carbohydrate, protein, and fat intakes were estimated as absolute intake (g/day) and percentage of total energy (% of total energy). Protein and fat intakes were divided into plant or animal protein and fat according to food sources. The sources of plant protein and fat were plant foods, including beans, nuts, grains, and vegetables; the sources of animal protein and fat were animal foods including meat (beef, pork, poultry, and other), fish (excluding seaweeds), eggs, and milk and dairy products (yogurt, cheese, and other). Based on the 2015 Dietary Reference Intakes for Koreans (KDRIs) [20], energy intake was compared with the estimated energy requirement (EER) to evaluate the adequacy of energy intake.

Food items consumed were categorized into grains (300 kcal/serving); meat, fish, eggs, and beans (MFEB) (100 kcal/serving); vegetables (15 kcal/serving); fruits (50 kcal/serving); and milk and dairy products (125 kcal/serving). Food group consumption was calculated as the number of servings per day. Food group consumption was evaluated compared with the recommended number of servings for each food group based on the recommended dietary pattern by life stage of the KDRIs [20].

### Definitions of moderate- and high-carbohydrate diets

A moderate-carbohydrate diet with plant protein intake was defined based on the 211 Diet, a balanced meal plan developed by Pulmuone (Seoul, Korea). The 211 Diet emphasizes the selection of protein foods with low saturated fat, fresh vegetables, and whole grains to prevent excessive carbohydrate consumption. At the nutrient level, the 211 diet recommends consuming 50% of daily energy from carbohydrates and higher plant protein, compared with animal protein, which can be defined using the ratio of plant protein to animal protein. Considering the overall high carbohydrate intake in Korean adults [9], moderate- and high-carbohydrate diets were defined as 50–60% and ≥ 70%, respectively. Thus, four types of diets were defined according to carbohydrate and protein intake (Table 1): moderate-carbohydrate diet with plant protein (MCP), moderate-carbohydrate diet with animal protein (MCA), high-carbohydrate diet with plant protein (HCP), and high-carbohydrate diet with animal protein (HCA). If participants did not consume any animal protein, the ratio of plant to animal protein was regarded as ≥ 1. After exclusion of those who did not satisfy the criteria of these moderate- and high-carbohydrate diets, 7965 participants (3196 men and 4769 women) were selected for the final analysis.

**Table 1:** Moderate- and high-carbohydrate diets by protein source

Diet	Carbohydrate intake (% of energy)	Ratio of plant to animal protein
Moderate-carbohydrate diet with plant protein	50–60%	≥ 1
Moderate-carbohydrate diet with animal protein	50–60%	< 1
High-carbohydrate diet with plant protein	≥ 70%	≥ 1
High-carbohydrate diet with animal protein	≥ 70%	< 1

### Measurement of anthropometric and biochemical variables

Anthropometric variables (weight, height, and waist circumference), blood pressure, and biochemical variables (total cholesterol, HDL-cholesterol, triglycerides, and fasting glucose levels) were measured at a mobile examination center using standardized procedures and calibrated equipment. Body mass index (BMI) was calculated based on weight and height (kg/m^2^). Systolic and diastolic blood pressure (SBP and DBP) were measured three times and the average of the second and third readings was used. Blood indices of participants who fasted for at least 8 h were used. Low-density lipoprotein (LDL)-cholesterol was calculated in accordance with the Friedewald formula only for participants whose triglyceride level was < 400 mg/dL [21]. The details of the laboratory procedures are available elsewhere [22].

### Cardiovascular risk factors

Cardiovascular risk factors assessed in this study were dyslipidemia, metabolic syndrome, hypertension, and type 2 diabetes. Dyslipidemia, which comprises several lipid abnormalities, was diagnosed as follows in accordance with the Korean Guidelines for the Management of Dyslipidemia (4th edition) [23]: 1) elevated total cholesterol (≥ 240 mg/dL), 2) elevated triglycerides (≥ 200 mg/dL), 3) elevated LDL-cholesterol (≥ 160 mg/dL), and 4) reduced HDL-choletserol (< 40 mg/dL). Based on the National Cholesterol Education Program Adult Treatment Panel III criteria [24], with the exception of waist circumference [25], metabolic syndrome was defined as the presence of three or more of the following metabolic abnormalities: 1) increased waist circumference (≥ 90 cm for men and ≥ 85 cm for women), 2) elevated blood pressure (SBP ≥ 130 mmHg or DBP ≥ 85 mmHg), 3) reduced HDL-cholesterol (< 40 mg/dL for men and < 50 mg/dL for women), 4) elevated triglycerides (≥ 150 mg/dL), and 5) elevated fasting glucose (≥ 100 mg/dL). Hypertension was defined as an SBP of ≥ 140 mmHg or a DBP of ≥ 90 mmHg and type 2 diabetes as a fasting glucose level of ≥ 126 mg/dL.

### Assessment of confounders

Sociodemographic characteristics (e.g., age, sex, education level, and household income) and health behaviors (e.g., physical activity, alcohol consumption, and smoking) were assessed in a health interview at the mobile examination center and were included as potential confounders. Education level was classified into middle school or lower, high school, or college or higher. Household income was divided into lowest, lower middle, upper middle, or highest quartile of monthly household income. Physical activity was defined as “yes,” if participants performed vigorous-intensity activities for at least 75 min, or moderate-intensity activities for at least 150 min, or an equivalent combination of moderate- and vigorous-intensity activity during a typical week. Alcohol consumption was categorized as none (no consumption of any type of alcoholic beverage or less than once per month over the past year), moderate (consumption of alcoholic beverages more than once per month over the past year), and high (consumption of more than seven glasses of alcoholic beverages for men and five glasses of alcoholic beverages for women per occasion more than two times per week). Smoking status was classified as never (never smoked cigarettes or smoked < 100 cigarettes in their lifetime), former (smoked ≥ 100 cigarettes in their lifetime but currently not smoking), and current (smoked ≥ 100 cigarettes in their lifetime and currently smoking). Survey period, BMI, and total energy intake were also considered as confounding variables.

### Statistical analysis

Statistical analysis was carried out using SAS software (ver. 9.4; SAS Institute, Cary, NC, USA). The complex sampling design parameters of KNHANES such as strata, cluster, and weight were used in the PROC SURVEY procedures to represent the Korean population. All *p*-values were two sided and a *p*-value < 0.05 was considered indicative of statistical significance.

Continuous variables are presented as means ± standard errors (SEs) and categorical variables are presented as numbers (percentages). Continuous variables were log-transformed to normalize their distributions; these values were used for statistical tests. Differences in general characteristics among the diets were examined using the chi-square test. Adjusted mean values of nutrient and food group intakes were calculated using a general linear model after adjustment for confounders. Multiple logistic regression analysis was performed to estimate the odds ratios (ORs) and 95% confidence intervals (CIs) of cardiovascular risk factors according to sex with a moderate-carbohydrate diet with plant protein (MCP) set as a reference after adjustment for confounders. This analysis was further stratified according to age group (19–49 and ≥ 50 years) for each sex. In the age-stratified analysis, type 2 diabetes was excluded due to the small number of cases.

## Results

### General characteristics of the participants

The distribution of sociodemographic characteristics and health behaviors differed among the diets (Table 2). Participants with a high-carbohydrate diet (≥ 70% of energy) were older than those with a moderate-carbohydrate diet (50–60% of energy) (*p* < 0.0001). Education level and household income tended to be lower in the high than the moderate carbohydrate diet groups (*p* < 0.0001 for all); they were lowest in participants with a high-carbohydrate diet with plant protein (HCP). The proportion of participants who performed physical activity was highest among those with a moderate-carbohydrate diet with plant protein (MCP) and lowest among those with an HCP (*p* < 0.0001). Participants who consumed more animal than plant protein were more likely to drink alcohol frequently, compared with those who consumed more plant than animal protein (*p* < 0.0001). The proportion of current smokers was lowest among participants with an HCP (*p* < 0.0001).

**Table 2:** General characteristics of the participants according to moderate- and high-carbohydrate diets, stratified by protein source

n, (%)^**a**^	Moderate carbohydrate diet with plant protein (MCP)^**b**^ (***n*** = 981)	Moderate carbohydrate diet with animal protein (MCA)^**b**^ (***n*** = 2209)	High carbohydrate diet with plant protein (HCP)^**b**^ (***n*** = 4283)	High carbohydrate diet with animal protein (HCA)^**b**^ (***n*** = 492)	***P*** value
Sex					< 0.0001
Male	392 (50.8)	992 (54.3)	1610 (45.1)	202 (50.2)	
Female	589 (49.2)	1217 (45.7)	2673 (54.9)	290 (49.8)	
Age group (years)					< 0.0001
19–29	230 (32.1)	541 (32.7)	328 (13.2)	85 (24.5)	
30–49	517 (49.6)	1221 (52.1)	1441 (39.3)	201 (42.9)	
50–64	189 (15.6)	354 (13.0)	1458 (31.8)	140 (24.1)	
≥ 65	45 (2.7)	93 (2.3)	1056 (15.7)	66 (8.5)	
Survey period					< 0.0001
2013–2015	552 (57.5)	1204 (55.6)	2649 (62.8)	277 (58.2)	
2016–2017	429 (42.5)	1005 (44.4)	1634 (37.2)	215 (41.8)	
Education					< 0.0001
Middle school or lower	101 (8.2)	186 (7.0)	1595 (28.9)	107 (16.2)	
High school	362 (40.1)	864 (41.5)	1364 (36.8)	177 (39.9)	
College or higher	502 (51.7)	1115 (51.4)	1227 (34.3)	195 (43.9)	
Household income					< 0.0001
Lowest	67 (7.0)	163 (7.4)	889 (16.8)	67 (11.9)	
Lower middle	233 (23.4)	478 (21.6)	1164 (26.8)	117 (22.3)	
Upper middle	335 (34.8)	757 (34.6)	1131 (28.6)	142 (29.6)	
Highest	343 (34.7)	807 (36.4)	1089 (27.8)	164 (36.3)	
Physical activity, yes^c^	529 (58.7)	1106 (54.6)	1811 (46.9)	243 (53.5)	< 0.0001
Alcohol consumption^d^					< 0.0001
None	356 (32.3)	745 (30.9)	2279 (48.6)	210 (37.5)	
Moderate	499 (53.5)	1120 (52.5)	1674 (43.6)	224 (49.4)	
High	123 (14.2)	334 (16.6)	282 (7.9)	54 (13.1)	
Smoking^e^					< 0.0001
Never	638 (59.7)	1406 (60.1)	2897 (64.2)	311 (59.3)	
Former	141 (14.8)	321 (14.4)	726 (17.7)	81 (16.9)	
Current	199 (25.6)	472 (25.4)	606 (18.1)	95 (23.8)	

^a^ Values are presented as the weighted percentages of the participants using the complex sampling design parameters of KNHANES

^b^ MCP = carbohydrate intake 50–60% of energy + plant/animal protein ≥ 1; MCA = carbohydrate intake 50–60% of energy + plant/animal protein < 1; HCP = carbohydrate intake ≥ 70% of energy + plant/animal protein ≥ 1; HCA = carbohydrate intake ≥ 70% of energy + plant/animal protein < 1

^c^ Physical activity: “yes”, performed vigorous-intensity activities for at least 75 min, or moderate-intensity activities for at least 150 min, or an equivalent combination of moderate- and vigorous-intensity activity during a typical week

^d^ Alcohol consumption: “none”, no consumption of any type of alcoholic beverage or drank less than once per month over the past year, “moderate”, consumption of alcoholic beverages more than once per month over the past year, “high”, consumption of more than seven glasses of alcoholic beverages for men and five glasses of alcoholic beverages for women per occasion more than two times per week

^e^ Smoking: “never”, never smoked cigarettes of smoked < 100 cigarettes in their lifetime, “former”, smoked ≥ 100 cigarettes in their lifetime but currently not smoking, “current”, smoked ≥ 100 cigarettes in their lifetime and currently smoking

### Dietary intakes

Energy intake was higher in the moderate than the high-carbohydrate diet groups (*p* < 0.0001) (Table 3). However, the energy intake of the moderate-carbohydrate diet groups was at a satisfactory level, compared with the EER. Dietary fiber intake was higher in participants with higher intake of plant protein, compared with participants with higher intake of animal protein (*p* < 0.0001).

**Table 3:** Energy and macronutrient intakes according to moderate- and high-carbohydrate diets, stratified by protein source

	Moderate carbohydrate diet with plant protein (MCP)^**a**^ (***n*** = 981)	Moderate carbohydrate diet with animal protein (MCA)^**a**^ (***n*** = 2209)	High carbohydrate diet with plant protein (HCP)^**a**^ (***n*** = 4283)	High carbohydrate diet with animal protein (HCA)^**a**^ (***n*** = 492)	***P*** value^**b**^
Energy (kcal)	2170.7 ± 29.9^c^	2235.3 ± 22.1	1871.6 ± 18.2	1783.2 ± 38.2	< 0.0001
Energy (% of EER)	98.4 ± 1.3	101.1 ± 0.9	85.9 ± 0.8	82.2 ± 1.7	< 0.0001
Carbohydrate (g)	252.0 ± 1.8	238.2 ± 1.5	348.1 ± 1.5	329.6 ± 2.8	< 0.0001
Protein (g)	64.8 ± 0.7	83.5 ± 0.7	55.2 ± 0.4	68.1 ± 0.7	< 0.0001
Plant protein	42.3 ± 0.5	28.2 ± 0.3	38.4 ± 0.3	30.0 ± 0.4	< 0.0001
Animal protein	22.4 ± 0.5	55.2 ± 0.6	16.7 ± 0.3	37.9 ± 0.5	< 0.0001
Fat (g)	62.1 ± 0.6	53.8 ± 0.4	24.7 ± 0.3	23.9 ± 0.6	< 0.0001
Plant fat	43.5 ± 0.7	23.6 ± 0.4	16.7 ± 0.3	12.1 ± 0.5	< 0.0001
Animal fat	18.6 ± 0.5	30.1 ± 0.4	8.0 ± 0.2	11.9 ± 0.4	< 0.0001
Dietary fiber (g)	23.8 ± 0.5	19.7 ± 0.3	24.9 ± 0.3	21.6 ± 0.6	< 0.0001
Energy from (%)					
Carbohydrate	56.9 ± 0.1	56.1 ± 0.1	76.3 ± 0.1	73.5 ± 0.1	< 0.0001
Protein	14.2 ± 0.1	18.1 ± 0.1	11.9 ± 0.1	15.1 ± 0.1	< 0.0001
Fat	28.9 ± 0.2	25.8 ± 0.1	11.8 ± 0.1	11.4 ± 0.2	< 0.0001
SFA	8.5 ± 0.1	7.7 ± 0.1	3.5 ± 0.0	3.4 ± 0.1	< 0.0001
MUFA	9.3 ± 0.1	8.4 ± 0.1	3.3 ± 0.0	3.2 ± 0.1	< 0.0001
PUFA	7.4 ± 0.1	6.1 ± 0.1	3.1 ± 0.0	2.9 ± 0.1	< 0.0001

*Abbreviations*: *EER* Estimated energy requirement, *MUFA* Monounsaturated fatty acids, *PUFA* Polyunsaturated fatty acids, *SFA* Saturated fatty acids

^a^MCP = carbohydrate intake 50–60% of energy + plant/animal protein ≥ 1; MCA = carbohydrate intake 50–60% of energy + plant/animal protein < 1; HCP = carbohydrate intake ≥ 70% of energy + plant/animal protein ≥ 1; HCA = carbohydrate intake ≥ 70% of energy + plant/animal protein < 1

^b^Log-transformed values were used for statistical tests

^c^Values are presented as means ± SEs after adjustment for age, sex, body mass index, education, household income, physical activity, smoking, survey period, alcohol consumption, and total energy intake (except the model of energy intake)

The grain intake of the high-carbohydrate diet with plant protein (HCP) group was highest (*p* < 0.0001) and was approximately 115% of the recommended dietary pattern (Table 4). The MFEB and grain intakes of the moderate-carbohydrate diet with plant protein (MCP) group were 81.2 and 96.0% of the recommended level, respectively; however, the fruit intake was 52.6% of the recommended dietary pattern, which was lower than that of the high-carbohydrate diet groups.

**Table 4:** Food group consumption according to moderate- and high-carbohydrate diets, stratified by protein source

	Moderate carbohydrate diet with plant protein (MCP)^**a**^ (***n*** = 981)	Moderate carbohydrate diet with animal protein (MCA)^**a**^ (***n*** = 2209)	High carbohydrate diet with plant protein (HCP)^**a**^ (***n*** = 4283)	High carbohydrate diet with animal protein (HCA)^**a**^ (***n*** = 492)	***P*** value^**b**^
No of servings					
Grain	3.3 ± 0.0^c^	2.4 ± 0.0	4.0 ± 0.0	3.3 ± 0.1	< 0.0001
MFEB	3.5 ± 0.1	5.3 ± 0.1	1.8 ± 0.0	2.8 ± 0.1	< 0.0001
Meat	1.6 ± 0.1	3.4 ± 0.1	0.7 ± 0.0	1.2 ± 0.1	< 0.0001
Fish	0.4 ± 0.0	0.9 ± 0.0	0.4 ± 0.0	1.1 ± 0.0	< 0.0001
Eggs	0.4 ± 0.0	0.5 ± 0.0	0.3 ± 0.0	0.3 ± 0.0	< 0.0001
Beans	1.1 ± 0.1	0.5 ± 0.0	0.4 ± 0.0	0.3 ± 0.0	< 0.0001
Vegetables	6.3 ± 0.2	6.5 ± 0.1	6.7 ± 0.1	6.0 ± 0.2	0.0102
Fruits	1.0 ± 0.1	1.2 ± 0.1	2.2 ± 0.1	2.6 ± 0.2	< 0.0001
Milk and dairy products	0.5 ± 0.0	0.6 ± 0.0	0.3 ± 0.0	0.5 ± 0.1	< 0.0001
% Servings^d^					
Grain	96.0 ± 1.3	69.6 ± 0.9	114.6 ± 0.8	94.9 ± 1.4	< 0.0001
MFEB	81.2 ± 1.8	120.5 ± 1.1	41.2 ± 0.7	64.8 ± 1.2	< 0.0001
Vegetables	80.3 ± 2.3	82.2 ± 1.4	85.0 ± 1.3	76.7 ± 2.4	0.0115
Fruits	52.6 ± 3.8	61.8 ± 3.1	102.3 ± 3.1	121.2 ± 6.5	< 0.0001
Milk and dairy products	45.6 ± 3.7	62.8 ± 3.0	28.9 ± 2.0	53.3 ± 5.8	< 0.0001

*Abbreviations*: *MFEB* Meat, fish, eggs, and beans

^a^MCP = carbohydrate intake 50–60% of energy + plant/animal protein ≥ 1; MCA = carbohydrate intake 50–60% of energy + plant/animal protein < 1; HCP = carbohydrate intake ≥ 70% of energy + plant/animal protein ≥ 1; HCA = carbohydrate intake ≥ 70% of energy + plant/animal protein < 1

^b^Log-transformed values were used for statistical tests

^c^Values are presented as means ± SEs after adjustment for age, sex, body mass index, education, household income, physical activity, smoking, survey period, alcohol consumption, and total energy intake

^d^%Servings = the number of servings consumed ÷ recommended number of servings based on Dietary Reference Intakes for Koreans × 100

### Association of moderate- and high-carbohydrate diets with cardiovascular risk factors

Table 5 presents the multivariable-adjusted ORs and 95% CIs of cardiovascular risk factors according to sex. In men, the high-carbohydrate diet with plant protein (HCP) and with animal protein (HCA) groups had higher ORs for an elevated total cholesterol level, reduced HDL-cholesterol level, and metabolic syndrome, compared with the moderate-carbohydrate diet with plant protein (MCP) group; HCP was associated with increased ORs for an elevated triglyceride level (OR, 1.41; 95% CI, 1.04–1.91) and an elevated fasting glucose level (OR, 1.41; 95% CI, 1.02–1.95), compared with MCP. Men in the moderate-carbohydrate diet with animal protein (MCA) group had higher ORs for an elevated total cholesterol level (OR, 1.80; 95% CI, 1.17–3.04), reduced HDL-cholesterol level (OR, 1.54; 95% CI, 1.07–2.22), and metabolic syndrome (OR, 1.51; 95% CI, 1.02–2.22) than men in the MCP group. In women, only the HCP group had an increased OR for a reduced HDL-cholesterol level (OR, 1.30; 95% CI, 1.03–1.65), compared with the MCP group.

**Table 5:** Multivariable-adjusted odds ratios and 95% confidence intervals of cardiovascular risk factors according to moderate- and high-carbohydrate diets, stratified by protein source

**Men**	**Moderate carbohydrate diet with plant protein (MCP)**^**a**^ **(*n*** **= 392)**	**Moderate carbohydrate diet with animal protein (MCA)**^**a**^ **(*n*** **= 992)**	**High carbohydrate diet with plant protein (HCP)**^**a**^ **(*n*** **= 1610)**	**High carbohydrate diet with animal protein (HCA)**^**a**^ **(*n*** **= 202)**
Dyslipidemia				
Elevated total cholesterol	1.00^b^	1.80 (1.07–3.04)	1.94 (1.15–3.25)	2.11 (1.04–4.30)
Elevated triglycerides	1.00	0.92 (0.65–1.30)	1.05 (0.74–1.48)	0.90 (0.52–1.55)
Elevated LDL-cholesterol	1.00	1.48 (0.81–2.71)	1.77 (0.95–3.29)	1.93 (0.87–4.29)
Reduced HDL-cholesterol	1.00	1.54 (1.07–2.22)	1.53 (1.08–2.18)	2.66 (1.62–4.36)
Metabolic syndrome	1.00	1.51 (1.02–2.22)	1.73 (1.16–2.59)	2.42 (1.41–4.15)
Increased waist circumference	1.00	1.14 (0.84–1.55)	0.96 (0.71–1.30)	1.21 (0.77–1.92)
Elevated blood pressure	1.00	0.85 (0.64–1.13)	1.04 (0.77–1.39)	0.76 (0.49–1.17)
Reduced HDL-cholesterol	1.00	1.54 (1.07–2.22)	1.53 (1.08–2.18)	2.66 (1.62–4.36)
Elevated triglycerides	1.00	1.31 (0.99–1.75)	1.41 (1.04–1.91)	1.34 (0.86–2.08)
Elevated fasting glucose	1.00	1.26 (0.90–1.75)	1.41 (1.02–1.95)	1.37 (0.86–2.18)
Hypertension	1.00	1.02 (0.68–1.52)	1.25 (0.83–1.89)	0.86 (0.49–1.53)
Type 2 diabetes	1.00	0.93 (0.40–2.20)	1.34 (0.61–2.95)	1.47 (0.51–4.21)
**Women**	**MCP (*n*** **= 589)**	**MCA (*n*** **= 1217)**	**HCP (*n*** **= 2673)**	**HCA (*n*** **= 290)**
Dyslipidemia				
Elevated total cholesterol	1.00	1.25 (0.79–1.99)	1.38 (0.88–2.17)	1.51 (0.80–2.86)
Elevated triglycerides	1.00	1.15 (0.69–1.91)	1.00 (0.63–1.60)	1.50 (0.74–3.01)
Elevated LDL-cholesterol	1.00	1.08 (0.67–1.74)	1.20 (0.76–1.89)	1.04 (0.52–2.08)
Reduced HDL-cholesterol	1.00	1.05 (0.66–1.68)	1.28 (0.82–2.00)	0.88 (0.46–1.68)
Metabolic syndrome	1.00	0.97 (0.61–1.54)	1.06 (0.69–1.62)	1.14 (0.64–2.06)
Increased waist circumference	1.00	0.98 (0.70–1.37)	1.03 (0.77–1.39)	0.76 (0.48–1.23)
Elevated blood pressure	1.00	1.24 (0.85–1.83)	1.10 (0.76–1.57)	1.40 (0.88–2.22)
Reduced HDL-cholesterol	1.00	1.12 (0.89–1.43)	1.30 (1.03–1.65)	1.01 (0.70–1.46)
Elevated triglycerides	1.00	0.87 (0.61–1.23)	0.89 (0.64–1.23)	1.02 (0.64–1.63)
Elevated fasting glucose	1.00	1.33 (0.97–1.83)	1.22 (0.90–1.64)	1.20 (0.77–1.85)
Hypertension	1.00	1.23 (0.69–2.22)	1.21 (0.72–2.03)	1.60 (0.79–3.25)
Type 2 diabetes	1.00	1.41 (0.62–3.21)	0.79 (0.36–1.75)	1.46 (0.49–4.34)

*Abbreviations*: *HDL* High-density lipoprotein-cholesterol, *LDL* Low-density lipoprotein-cholesterol

^a^ MCP = carbohydrate intake 50–60% of energy + plant/animal protein ≥ 1; MCA = carbohydrate intake 50–60% of energy + plant/animal protein < 1; HCP = carbohydrate intake ≥ 70% of energy + plant/animal protein ≥ 1; HCA = carbohydrate intake ≥ 70% of energy + plant/animal protein < 1

^b^ Adjusted for age, body mass index (except the model of waist circumference), education, household income, physical activity, smoking, survey period, alcohol consumption, and total energy intake

Considering the age differences among participants on the four types of diets, an age-stratified analysis was conducted for each sex (Table 6, Supplementary Table 1). In younger adults aged 19–49 years, most significant associations were maintained in men and women, except for elevated total cholesterol in men with an HCA (Table 6). Younger women with an MCA (OR, 2.21; 95% CI, 1.11–4.38) and those with an HCP (OR, 2.38; 95% CI, 1.20–4.73) also had increased ORs for an elevated total cholesterol level, compared with those with an MCP. Younger women who had an HCP also exhibited a higher OR for an elevated fasting glucose level (OR, 1.57; 95% CI, 1.07–2.29), compared with younger women who had an MCP. In contrast, among adults aged ≥ 50 years, no significant associations were found in men, whereas several cardiovascular risk factors were associated with diets in women (Supplementary Table 1). Compared with the MCP group, the MCA group had higher ORs for metabolic syndrome including reduced HDL-cholesterol; the HCP and HCA groups had higher ORs for hypertension in older women.

**Table 6:** Multivariable-adjusted odds ratios and 95% confidence intervals of cardiovascular risk factors according to moderate- and high-carbohydrate diets, stratified by protein source in younger adults (aged 19–49 years)

**Men**	**Moderate carbohydrate diet with plant protein (MCP)**^**a**^ **(*n*** **= 295)**	**Moderate carbohydrate diet with animal protein (MCA)**^**a**^ **(*n*** **= 774)**	**High carbohydrate diet with plant protein (HCP)**^**a**^ **(*n*** **= 606)**	**High carbohydrate diet with animal protein (HCA)**^**a**^ **(*n*** **= 112)**
Dyslipidemia				
Elevated total cholesterol	1.00^b^	2.22 (1.16–4.24)	2.57 (1.34–4.91)	2.56 (1.00–6.56)
Elevated triglycerides	1.00	1.04 (0.70–1.55)	1.10 (0.72–1.67)	1.00 (0.50–2.02)
Elevated LDL-cholesterol	1.00	2.20 (0.96–5.04)	3.02 (1.33–6.87)	2.55 (0.82–7.97)
Reduced HDL-cholesterol	1.00	1.75 (1.15–2.67)	1.92 (1.26–2.92)	3.69 (1.98–6.89)
Metabolic syndrome	1.00	2.07 (1.29–3.32)	2.97 (1.76–5.03)	3.96 (1.87–8.40)
Increased waist circumference	1.00	1.13 (0.80–1.60)	0.91 (0.64–1.31)	1.34 (0.77–2.33)
Elevated blood pressure	1.00	0.84 (0.60–1.19)	1.29 (0.89–1.86)	0.81 (0.45–1.45)
Reduced HDL-cholesterol	1.00	1.75 (1.15–2.67)	1.92 (1.26–2.92)	3.69 (1.98–6.89)
Elevated triglycerides	1.00	1.42 (1.02–1.98)	1.61 (1.12–2.31)	1.46 (0.83–2.57)
Elevated fasting glucose	1.00	1.35 (0.89–2.06)	2.06 (1.34–3.18)	1.94 (1.02–3.69)
Hypertension	1.00	1.01 (0.63–1.64)	1.72 (1.03–2.87)	1.01 (0.45–2.25)
**Women**	**MCP (*n*** **= 452)**	**MCA (*n*** **= 988)**	**HCP (*n*** **= 1163)**	**HCA (*n*** **= 174)**
Dyslipidemia				
Elevated total cholesterol	1.00	2.21 (1.11–4.38)	2.38 (1.20–4.73)	2.02 (0.75–5.41)
Elevated triglycerides	1.00	0.94 (0.50–1.76)	0.88 (0.47–1.66)	1.18 (0.47–2.98)
Elevated LDL-cholesterol	1.00	1.76 (0.84–3.70)	2.07 (0.99–4.31)	1.08 (0.31–3.78)
Reduced HDL-cholesterol	1.00	1.07 (0.62–1.86)	1.52 (0.89–2.61)	1.34 (0.64–2.79)
Metabolic syndrome	1.00	0.69 (0.36–1.29)	0.95 (0.51–1.77)	0.81 (0.36–1.81)
Increased waist circumference	1.00	0.98 (0.66–1.45)	1.09 (0.76–1.58)	0.88 (0.47–1.67)
Elevated blood pressure	1.00	1.36 (0.81–2.27)	1.14 (0.67–1.96)	1.68 (0.90–3.17)
Reduced HDL-cholesterol	1.00	1.01 (0.77–1.34)	1.37 (1.03–1.81)	0.94 (0.60–1.48)
Elevated triglycerides	1.00	0.76 (0.49–1.17)	0.85 (0.55–1.31)	0.88 (0.47–1.66)
Elevated fasting glucose	1.00	1.29 (0.87–1.91)	1.57 (1.07–2.29)	1.26 (0.70–2.27)
Hypertension	1.00	1.23 (0.56–2.72)	0.92 (0.41–2.09)	1.36 (0.44–4.21)

*Abbreviations*: *HDL* High-density lipoprotein-cholesterol, *LDL* Low-density lipoprotein-cholesterol

^a^ MCP = carbohydrate intake 50–60% of energy + plant/animal protein ≥ 1; MCA = carbohydrate intake 50–60% of energy + plant/animal protein < 1; HCP = carbohydrate intake ≥ 70% of energy + plant/animal protein ≥ 1; HCA = carbohydrate intake ≥ 70% of energy + plant/animal protein < 1

^b^ Adjusted for body mass index (except the model of waist circumference), education, household income, physical activity, smoking, survey period, alcohol consumption, and total energy intake

## Discussion

This large-scale cross-sectional study of Korean adults compared associations of cardiovascular risk factors with moderate- and high-carbohydrate diets according to protein source. It showed that a moderate-carbohydrate diet with plant protein (MCP) was inversely associated with several cardiovascular risk factors, including elevated total cholesterol, reduced HDL-cholesterol, and elevated fasting glucose, especially in younger men and women.

Early studies reported inconsistent associations of dietary carbohydrate with cardiovascular risk factors between Asian and Western populations [26–31], which might be attributable to different ranges of carbohydrate intakes between these populations. A recent cross-sectional study reported that Korean adults consume more carbohydrates than US adults; a stronger association of dietary carbohydrate with metabolic syndrome was found in Korean adults [32]. Seidelmann et al. [12] reported a U-shaped association of carbohydrate intake with mortality, whereby Asian populations represented the right side of the curve and North American and European populations the left side. Therefore, nutritional targets for preventing cardiovascular risk factors should be differentiated by considering different ranges of carbohydrate intake that are typical of Asian and Western countries. For Asian populations whose diets are typically high in carbohydrate, it is important to reduce the carbohydrate intake to a moderate level.

To lower carbohydrate intake, both protein and fat intakes can be increased without restriction or protein intake can be increased while allowing fat intake to reach an appropriate level [11]. Substitution of carbohydrates for dietary protein reduces the postprandial glycemic response [33, 34]. Although associations between dietary protein and cardiovascular risks have not been investigated in detail, a study of Asian Indians reported that a high-protein diet (29% of energy from protein) for 3 months promoted weight loss, compared with a standard diet (15% of energy from protein) in overweight and obese adults [35].

In this study, participants with a moderate carbohydrate diet with animal protein (MCA) had higher ORs for lipid abnormalities and metabolic syndrome compared with those with a moderate carbohydrate diet with plant protein (MCP), except among older adult men. These findings were consistent with the results of previous prospective cohort studies, which reported differences in associations of cardiovascular risks with animal- or plant-based low-carbohydrate diets in American adults [36–39]. Plant protein, especially soy protein, and the intake of isoflavone reportedly reduced the total and LDL-cholesterol levels compared with animal protein [16]; moreover, they showed beneficial associations with inflammation and oxidative stress [40].

Few studies of dietary pattern or soy protein intake in Koreans have been conducted. A dietary pattern including high intake of whole grains and beans was associated with a lower risk of insulin resistance in Korean adults [41]. Additionally, a prospective cohort study reported that soy protein and isoflavone intake were related to a reduced risk of metabolic syndrome among middle-aged Korean adults [42].

Further studies are needed to optimize protein intake along with food source in moderate-carbohydrate diets in Koreans. The findings of this study indicate that adequate intake of plant protein with a moderate carbohydrate intake (50–60% of energy) may ameliorate metabolic diseases. Further studies are warranted to confirm the effects of this type of diet on cardiovascular risk factors in Koreans.

This study had several limitations. First, its cross-sectional design prevented investigation of the causal relationship of a moderate- or high-carbohydrate diet with cardiovascular risk factors according to source of protein. Further clinical trials are needed to verify the effects of a moderate-carbohydrate diet with plant protein (MCP) on cardiovascular risk factors in Koreans. Second, dietary intake was measured using 1-day 24-h dietary recall, which might not represent the usual intake of participants. Third, participants were grouped based on their dietary intake, which caused the number of participants to differ among the groups. Thus, caution is required when interpreting the results, especially for older adults. Fourth, the study did not consider fat intake. However, a previous study defined a low-carbohydrate diet using all macronutrients; it showed that a low-carbohydrate diet was not associated with metabolic diseases, with the exception of a reduced HDL-cholesterol level, in Korean adults [43]. We propose that macronutrient composition is evaluated in various ways to develop effective dietary recommendations for prevention of cardiovascular diseases. Despite these limitations, to our knowledge, this study is the first to investigate associations of moderate- and high-carbohydrate diets with cardiovascular risk factors considering protein intake among Korean adults. Moreover, the finding that an MCP was inversely associated with cardiovascular risk factors was novel.

## Conclusions

A moderate-carbohydrate diet with a high intake of plant protein was inversely associated with cardiovascular risk factors, especially among younger Korean adults. Further intervention studies that consider protein sources and carbohydrate intake levels are required to develop a diet that enhances the cardiovascular health of Koreans.

## Supplementary information

**Additional file 1: Supplementary Table 1.** Multivariable-adjusted odds ratios and 95% confidence intervals of cardiovascular risk factors according to moderate- and high-carbohydrate diets, stratified by protein source in older adults (aged ≥ 50 years).

## Data Availability

The datasets used and/or analyzed during the current study are available in https://knhanes.cdc.go.kr/knhanes/eng/index.do.

## Abbreviations

BMI: Body mass index
CI: Confidence interval
DBP: Diastolic blood pressure
EER: Estimated energy requirement
HCA: High-carbohydrate diet with animal protein
HCP: High-carbohydrate diet with plant protein
HDL: High-density lipoprotein-cholesterol
KCDC: Korea Centers for Disease Control & Prevention
KDRI: Dietary Reference Intakes for Koreans
KNHANES: Korea National Health and Nutrition Examination Survey
LDL: Low-density lipoprotein-cholesterol
MCA: Moderate-carbohydrate diet with animal protein
MCP: Moderate-carbohydrate diet with plant protein
MFEB: Meat, fish, eggs, and beans
OR: Odds ratio
SBP: Systolic blood pressure
SE: Standard error
US: United States
