# Supplementary material for: A moderate-carbohydrate diet with plant protein is inversely associated with cardiovascular risk factors: the Korea National Health and Nutrition Examination Survey 2013–2017
Source: Nutr J. 2020 Aug 14;19:84. doi: 10.1186/s12937-020-00603-2 (PMC7427735; doi:10.1186/s12937-020-00603-2)
Supplement: Supplementary file 1 — Additional file 1: Supplementary Table 1. Multivariable-adjusted odds ratios and 95% confidence intervals of cardiovascular risk factors according to moderate- and high-carbohydrate diets, stratified by protein source in older adults (aged ≥ 50 years). [file 12937_2020_603_MOESM1_ESM.docx]

**Supplementary Table 1** Multivariable-adjusted odds ratios and 95% confidence intervals of cardiovascular risk factors according to moderate- and high-carbohydrate diets, stratified by protein source in older adults (aged ≥50 years)

| **Men** | **Moderate carbohydrate diet with plant protein (MCP) ^1^**  **(n = 97)** | **Moderate carbohydrate diet with animal protein (MCA) ^1^ (n = 218)** | **High carbohydrate diet with plant protein (HCP) ^1^**  **(n = 1004)** | **High carbohydrate diet with animal protein (HCA) ^1^**  **(n = 90)** |
| --- | --- | --- | --- | --- |
| Dyslipidemia |  |  |  |  |
| Elevated total cholesterol | 1.00 ^2^ | 0.94 (0.36–2.48) | 1.30 (0.55–3.05) | 1.41 (0.46–4.32) |
| Elevated triglycerides | 1.00 | 0.63 (0.32–1.26) | 1.09 (0.61–1.94) | 0.88 (0.36–2.16) |
| Elevated LDL-cholesterol | 1.00 | 0.58 (0.23–1.48) | 0.74 (0.32–1.71) | 1.11 (0.35–3.53) |
| Reduced HDL-cholesterol | 1.00 | 0.95 (0.47–1.89) | 0.96 (0.50–1.82) | 1.25 (0.56–2.81) |
| Metabolic syndrome | 1.00 | 0.79 (0.38–1.63) | 0.84 (0.44–1.61) | 1.18 (0.51–2.76) |
| Increased waist circumference | 1.00 | 1.16 (0.60–2.27) | 1.04 (0.57–1.92) | 1.11 (0.50–2.48) |
| Elevated blood pressure | 1.00 | 1.00 (0.56–1.79) | 0.98 (0.58–1.64) | 0.79 (0.39–1.60) |
| Reduced HDL-cholesterol | 1.00 | 0.95 (0.47–1.89) | 0.96 (0.50–1.82) | 1.25 (0.56–2.81) |
| Elevated triglycerides | 1.00 | 0.96 (0.54–1.71) | 1.11 (0.66–1.85) | 1.15 (0.56–2.34) |
| Elevated fasting glucose | 1.00 | 1.30 (0.71–2.39) | 1.10 (0.64–1.90) | 0.89 (0.43–1.86) |
| Hypertension | 1.00 | 1.06 (0.51–2.22) | 1.05 (0.55–2.00) | 0.79 (0.33–1.88) |
| **Women** | **MCP (n = 137)** | **MCA (n = 229)** | **HCP (n = 1510)** | **HCA (n = 116)** |
| Dyslipidemia |  |  |  |  |
| Elevated total cholesterol | 1.00 | 0.70 (0.35–1.40) | 0.99 (0.55–1.79) | 1.13 (0.49–2.60) |
| Elevated triglycerides | 1.00 | 2.20 (0.91–5.34) | 1.70 (0.75–3.85) | 2.58 (0.87–7.66) |
| Elevated LDL-cholesterol | 1.00 | 0.67 (0.34–1.34) | 0.84 (0.48–1.47) | 0.83 (0.34–2.00) |
| Reduced HDL-cholesterol | 1.00 | 1.04 (0.47–2.29) | 1.08 (0.55–2.12) | 0.32 (0.08–1.23) |
| Metabolic syndrome | 1.00 | 1.99 (1.01–3.93) | 1.82 (0.97–3.40) | 2.14 (0.95–4.82) |
| Increased waist circumference | 1.00 | 0.98 (0.54–1.78) | 1.20 (0.73–1.97) | 0.70 (0.35–1.39) |
| Elevated blood pressure | 1.00 | 1.04 (0.60–1.81) | 1.30 (0.80–2.10) | 1.34 (0.70–2.57) |
| Reduced HDL-cholesterol | 1.00 | 1.79 (1.05–3.05) | 1.53 (1.00–2.36) | 1.36 (0.72–2.57) |
| Elevated triglycerides | 1.00 | 1.37 (0.76–2.48) | 1.30 (0.80–2.13) | 1.54 (0.78–3.04) |
| Elevated fasting glucose | 1.00 | 1.59 (0.89–2.85) | 1.05 (0.63–1.77) | 1.12 (0.56–2.23) |
| Hypertension | 1.00 | 1.17 (0.52–2.60) | 2.00 (1.04–3.83) | 2.37 (1.02–5.51) |

^1^ MCP = carbohydrate intake 50–60% of energy + plant/animal protein ≥1; MCA = carbohydrate intake 50–60% of energy + plant/animal protein <1; HCP = carbohydrate intake ≥70% of energy + plant/animal protein ≥1; HCA = carbohydrate intake ≥70% of energy + plant/animal protein <1.

^2^ Adjusted for body mass index (except the model of waist circumference), education, household income, physical activity, smoking, survey period, alcohol consumption, and total energy intake.

Abbreviations: HDL: high-density lipoprotein-cholesterol; LDL: low-density lipoprotein-cholesterol.
